# Behind closed doors: Exploring the impact of COVID-19 related lockdown on domestic violence in Peru

**DOI:** 10.1016/j.ssmph.2023.101552

**Published:** 2023-11-11

**Authors:** Akram Hernández-Vásquez, Rodrigo Vargas-Fernández, Elena Tapia-López, Carlos Rojas-Roque

**Affiliations:** a Centro de Excelencia en Investigaciones Económicas y Sociales en Salud, Vicerrectorado de Investigación, Universidad San Ignacio de Loyola, Lima, Peru; b Universidad Científica del Sur, Lima, Peru; c HAMPI: Consultores en Salud, Lima, Peru; d Centre for Health Economics, University of York, Heslington, York, YO10 5DD, UK

**Keywords:** Pandemics, COVID-19, Hotlines, Domestic violence, Female, Peru

## Abstract

**Objectives:**

The COVID-19 pandemic and the lockdown measures implemented have generated an environment conducive to an increase in domestic violence. This study aimed to evaluate changes in calls reporting domestic violence to Línea 100 in Peru before, during and after strict lockdown, using a controlled interrupted time series analysis.

**Methods:**

Data from January 2018 to March 2022 from Línea 100, a national toll-free hotline service for survivors of domestic violence, were used. A quasi-experimental research design with controlled interrupted time series analysis was applied. The number of monthly calls reporting domestic violence was the outcome variable, while the sex of the callers was the treatment variable.

**Results:**

A significant increase in the number of calls was found during strict lockdown compared to the previous period. In addition, a decrease in the number of calls after confinement was observed. In all analyses, women were the most affected by domestic violence before, during and after lockdown.

**Conclusions:**

This study provides evidence on the impact of the COVID-19 pandemic on domestic violence in Peru. The findings highlight the need to strengthen domestic violence prevention and care services, especially during crisis situations such as the pandemic. Also, better targeted intervention strategies aimed at protecting women and promoting safe environments within the home are needed.

## Highlights

• Domestic violence is a pervasive social and health problem worldwide.
• Latin America and the Caribbean have witnessed a surge in domestic violence reports during the pandemic.
• The study evaluated changes in domestic violence calls in Peru before, during, and after strict lockdown.
• Findings revealed a significant increase in domestic violence calls during the strict lockdown period.

## 1 Introduction

Domestic violence is a social and health problem that occurs in the home or family and involves various types of violence such as child abuse, intimate partner violence, and violence inflicted on any member of the household regardless of gender (World Health Organization & Pan American Health Organization, 2012). Despite the eradication of all forms of violence being a sustainable development goal, it is estimated that more than one million women experienced violence by a current or former intimate partner at some point in their lives in 2018 (Sardinha et al., 2022), one billion children aged 2–17 years experienced some form of domestic violence in 2016 (World Health Organization, 2020), and more than nine thousand older adults experienced some form of domestic violence between 2002 and 2015 (Yon et al., 2017). The highest figures of domestic violence are observed in low- and middle-income countries (LMICs) (Garcia-Moreno et al., 2006), mainly due to the cultural context in which people live (example: "machismo") and the high rate of acceptance of violence (Sardinha & Nájera Catalán, 2018). Although worldwide figures of domestic violence reflect a large negative impact on people's health and well-being, periods of economic uncertainty, civil unrest, disasters and pandemics are associated with an even greater increase in violence experienced in the home (Lyons & Brewer, 2022).

The COVID-19 pandemic created a period of great uncertainty, which, in turn, generated an environment conducive to the development or increase of violent attitudes within the home through the preventive measures taken by 190 countries to control the spread of the disease (Phillips et al., 2021). Specifically, one of the measures that directly and indirectly influenced this socio-health problem is lockdown (which includes an stay-at-home order), which led to survivors of violence being isolated in the same home as their perpetrators (Lyons & Brewer, 2022). In fact, a systematic review (SR) that included data from 28 studies conducted in the Asia (n = 9 studies), North America (n = 13), Middle East (n = 2), Europe (n = 3) and Africa (n = 1) regions reported that the consequences of lockdown allowed perpetrators to exert more violence on their survivors due to economic dependence on the partner and increased stress (due to an increased unemployment rate, school and business closures) and alcohol consumption, control and isolation of the survivors due to lack of support networks, and underreporting of violence events in the homes (Letourneau et al., 2022). All these triggers in various regions of the world generated an increase in the prevalence of domestic violence (in all its forms) and its severity in various regions of the world (Peitzmeier et al., 2022; Thiel et al., 2022), with even new cases of domestic violence being reported with the initiation of preventive measures by COVID-19, as described in the biomedical literature.

There are many studies which point to different patterns in the reporting of domestic violence in the Latin American and Caribbean (LAC) region (De Souza Santos et al., 2022; Ortega Pacheco & Martínez Rudas, 2021). However, one study reported an increase in the reporting of this social problem in countries such as Argentina (+62%), Colombia (+98%) and Peru (+39%) with the onset of lockdown (Perez-Vincent & Carreras, 2022). Due to the increase in reported cases of domestic violence, governmental institutions in Mexico, Colombia, Argentina, Brazil, Ecuador and Chile created online and telephone strategies to report domestic violence and reduce its impact (Lima, 2020; Perez-Vincent & Carreras, 2022). In Peru, there are governmental and non-governmental organizations in charge of protecting the population from domestic violence, such as SOS Children's Villages Peru, CARE Peru, Ministry of Women and Vulnerable Populations (MIMP, by its acronym in Spanish), Defensoría del Pueblo, among others (CARE, 2023; Observatorio Nacional de la Violencia contra las Mujeres y los Integrantes del Grupo Familiar, 2023; SOS Children’s Villages, 2020). Particularly, the MIMP has Línea 100, which is a free public service that provides information, counseling, guidance and emotional support to people who are survivors of domestic violence (Ministerio de la Mujer y Poblaciones Vulnerable, 2021a, p. 100). This service can be used 24 h a day and has been a widely used strategy in the pandemic period due to the lack of availability of health and police services and the consequences of lockdown (Ministerio de la Mujer y Poblaciones Vulnerable, 2021a, p. 100).

Several institutions have estimated figures on the use of telephone calls to report domestic violence. The World Health Organization member states in Europe reported a 60% increase in emergency calls from women survivors of domestic violence in the pandemic period (Mahase, 2020). In addition, the World Bank noted an increase in telephone reports of violence against women in several countries in the LAC region, such as Mexico (+36%), Colombia (+91%), Peru (+48%) and Argentina (+32%), and even estimated an increase in femicides in Panama (+50%), Ecuador (+25%) and Costa Rica (+25%) during the period of lockdown (The World Bank, 2022). Specifically, in Peru, an official MIMP report showed an increase in the number of reports of domestic violence through telephone calls to Línea 100 during the pandemic (Programa Nacional Aurora, 2023), reflecting the current picture of the impact of the pandemic on domestic violence.

Although domestic violence is an urgent challenge in Peru, few studies have evaluated changes in this problem before and during lockdown (Agüero, 2021; Porter et al., 2021). Specifically, a study that used the Línea 100 data up to July 2020 reported an increase in the number of calls for domestic violence between the pre-pandemic and pandemic period (Agüero, 2021). However, evidence on the changes that this strategy has perceived in the periods before, during and after strict lockdown, and its variations by life stage and gender, is still scarce. Therefore, the present study aims to evaluate changes in calls to Línea 100 before, during and after strict lockdown among men and women and by life stage through a controlled interrupted time series analysis.

## 2 Materials and methods

### 2.1 Data source

This study used the data collected during the service of Línea 100 by the MIMP in the period between January 2018 and March 2022. Línea 100 is a national free telephone hotline service, provided on an uninterrupted basis for household members who are survivors of any act of violence (Ministerio de la Mujer y Poblaciones Vulnerable, 2021a). This service has been implemented since 2008 by the AURORA National Program of the MIMP with the aim of providing information, guidance, counseling and emotional support to people who are survivors of physical, psychological, sexual, economic or patrimonial violence. It also provides assistance to those who know of a case of abuse in their environment (Ministerio de la Mujer y Poblaciones Vulnerable, 2021a). Línea 100 acts as a referral center for the network of prevention, care and protection against violence, the cases of which are referred to the Urgent Care Service (SAU) and the Women's Emergency Centers (CEM) (Ministerio de la Mujer y Poblaciones Vulnerable, 2021a).

The telephone calls are taken by previously trained operators and are divided into three stages: i) call opening stage, which guarantees the entry of calls to the Línea 100 service through the provision of adequate telephone service and the reception of calls; ii) call development stage, which facilitates access to justice, protection and recovery of users through actions related to the identification, diagnosis and attention to the needs of individuals under ethical behaviors. When a case of risk is detected during this stage, coordination strategies are carried out with the Peruvian National Police, SAU and CEM; and iii) call completion stage, which allows clarifying the user's doubts and providing the necessary tools to solve the problematic situation. The information collected during the telephone call is placed on a telephone consultation record card by trained operators who ensure the data obtained remains confidential. This information includes the socio-demographic data of the informant or affected person, the reason for the consultation, data on the aggressors, risk or vulnerability assessment and actions taken to counteract or prevent the acts of violence (Ministerio de la Mujer y Poblaciones Vulnerable, 2021a). The operators have particular considerations during the telephone service in subpopulations that are at greater risk of violent acts (elderly, people with disabilities, minors, among others) (Ministerio de la Mujer y Poblaciones Vulnerable, 2021a). More information on processes, specifications and special cases of attention can be found in the Protocol of Attention Línea 100 (Ministerio de la Mujer y Poblaciones Vulnerable, 2021a).

### 2.2 Study population

The study population is comprised of individuals who have made a telephone call to Línea 100 reporting a domestic violence event.

### 2.3 Inclusion and exclusion criteria

Adult individuals aged 18 years or older, residing in urban or rural areas of Peru, who called Línea 100 between January 2018 and March 2022 were included. Individuals with incomplete information on the variables of interest in this study were excluded from this study.

### 2.4 Statistical analyses

In this study, a quasi-experimental research design was utilized, incorporating controlled interrupted time series (ITS) analysis. This approach allowed for comparisons of domestic violence calls before, during, and after a strict lockdown period within a well-defined timeframe (Lopez Bernal et al., 2016). By employing this statistical technique, the study aimed to estimate the impact of a policy change on a particular outcome of interest (Kontopantelis et al., 2015; Lopez Bernal et al., 2016).

The outcome variable of the study is a call reporting a domestic violence event. Calls were measured monthly. Men were defined as the control group while women were defined as the treatment group due to the fact that women have the highest rates of domestic violence in Peru (Centro Nacional de Epidemiologia, Prevencion y Control de Enfermedades, 2023). The intervention variable was defined as the start of the strict lockdown from March 2020 to July 2020. The strict lockdown was defined as the limitation to the exercise of the right to free transit of persons, with some exceptions such as the sale of food, medicines, medical services, fuel, police and military services and financial entities. In addition, the borders were temporarily closed (Presidencia del Consejo de Ministros, 2020). The study defined a second intervention variable, which was the post-strict lockdown and started from August 2020 and ended in March 2022.

The equation used to fit the data in the regression model was as follows:

(1) $$Y_{t}=\beta_{0}+\beta_{1}T_{t}+\beta_{2}X_{1t}+\beta_{3}X_{1t}T_{1t}+\beta_{4}Z+\beta_{5}{ZT}_{t}+\beta_{6}ZX_{1t}+\beta_{7}{ZX}_{1t}T_{t}+\beta_{8}X_{2t}T_{t}+\beta_{9}X_{2t}T_{2t}+\beta_{10}{ZX}_{2t}+\beta_{11}{ZX}_{2t}T_{2t}+\epsilon_{t}$$

In Equation (1), $Y_{t}$ is the aggregate violence calls measured each month t. $T_{t}$ represents the time, which is assessed every month, while $X_{1t}$ is the intervention variable, which represents the strict lockdown and is coded as 1 from March 2020 till July 2020 and is coded as zero otherwise. Z is the treatment variable and $X_{2t}$ is the second intervention variable, which represents the post-strict lockdown period and is coded as 1 from August 2022 till March 2022, and is otherwise coded as zero. The interaction terms among the variables mentioned above include $X_{1t}T_{1t}$, ${ZT}_{t}$, $ZX_{1t}$, ${ZX}_{1t}T_{t}$, $X_{2t}T_{t}$, $X_{2t}T_{2t}$, ${ZX}_{2t}$ and ${ZX}_{2t}T_{2t}$. The coefficients in this study are defined as follows: $\beta_{0}$ represents to the average violence calls per month for the control group at the beginning of the study, $\beta_{1}$ is the difference between the treatment and control group's pre-intervention trends, $\beta_{2}$ denotes the immediate change in the level of the violence call per month that takes place right after the implementation of the stringent lockdown, $\beta_{3}$ the control group's difference in pre-intervention to first-intervention trends, $\beta_{4}$ refers to the disparity in the level (intercept) of the violence calls per month between the treatment and control groups before the enforcement of the strict lockdown, $\beta_{5}$ the disparity between the treatment and control group's pre-intervention trends, $\beta_{6}$ signifies the disparity in the level of the violence calls per months between the treatment and control group right after the introduction of the intervention, $\beta_{7}$ represents the difference in trends between the treatment and control groups, specifically their variations from the pre-intervention period to the first intervention, $\beta_{8}$ represents the change in the level of the outcome that occurs in the period immediately following the introduction of the second intervention, $\beta_{9}$ indicates the variation in trends for the control group from the first to the second intervention, $\beta_{10}$ signifies the disparity in trends between the treatment and control groups, specifically their variations from the pre-second intervention period and $\beta_{11}$ represents the distinction in the trends between the treatment and control groups, specifically their variations from the first to the second intervention trends (Linden, 2017).

The ITS analysis was conducted using the Stata command “*itsa”* (Linden, 2015). To compare the characteristics between the control and treatment groups, the “*lincom*” command in Stata was employed. This command calculates point estimates, standard errors, t or z statistics, p-values, and confidence intervals (CIs) for linear combinations of coefficients following any estimation command (Linden, 2017).

In Equation (1) the autocorrelation was corrected using the lag 1 Newey-West standard errors (Harris & Tzavalis, 1999). The statistical software Stata v17 (Stata Corporation, College Station, Texas, USA) was utilized for all the analyses. In each analysis, statistical significance was assessed based on a *p*-value of less than or equal to 0.05. Subgroup analyses were performed according to the age of the individual who suffered the domestic violence. Specifically, we analyzed children (aged 5 years or below), adults (aged between 18 and 59 years old) and older adults (60 years old or older).

### 2.5 Ethical considerations

Since the study involved analyzing aggregated secondary data that is publicly available (https://portalestadistico.aurora.gob.pe/bases-de-datos-2022/) and does not allow for the identification of the evaluated participants, it did not necessitate the approval of an ethics committee.

## 3 Results

A total of 693,747 telephone call records to Línea 100 were included during the period from January 2018 to March 2022. In the general population, the highest average number of telephone call records to Línea 100 was observed during the strict lockdown (21,251.0; Standard deviation [SD]: 4520.5) as opposed to the periods before (8554.6; SD: 2472) and after lockdown (18,332.8; SD: 2371.0). This same pattern was observed in people belonging to the age groups 18 to 59 years and over 60 years, while in the population aged 0 to 17 years, the highest average number of telephone call reports to Línea 100 was found after the strict lockdown (Table 1).

**Table 1:** Mean monthly domestic violence calls to Linea 100 from January 2018 to March 2022.

	Domestic violence calls	
	Monthly mean	SD
Overall		
Before lockdown	8554.6	2472
During lockdown	21,251.0	4520.5
After lockdown	18,332.8	2371.0
0-17 years		
Before lockdown	3367.5	833.3
During lockdown	5458.3	1041
After lockdown	5517.8	506.1
18-59 years		
Before lockdown	4596.9	1443.8
During lockdown	13,539.7	3137.2
After lockdown	10,743.6	1711.1
60 or more years		
Before lockdown	456.1	162.5
During lockdown	1643.3	328.4
After lockdown	1448.4	222.9

SD: standard deviation.

Lockdown refers to the period of strict confinement that occurred between March and July 2020.

Strict lockdown started on March 16, 2020.

**Fig. 1:**
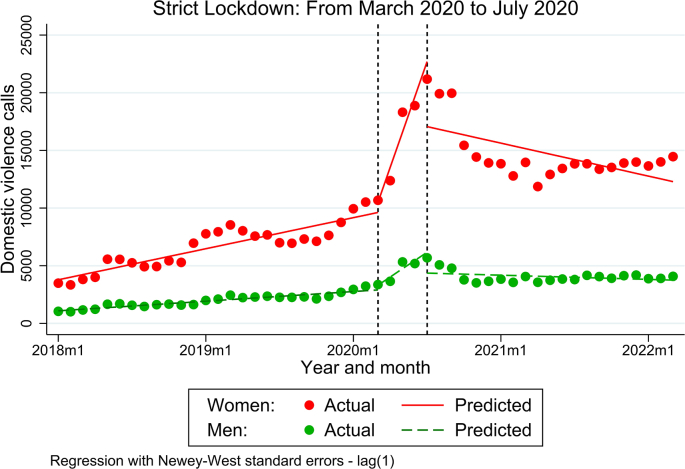
Interrupted Time Series: Domestic violence calls from January 2018 to March 2022.

Before the strict lockdown, domestic violence calls reported a slightly increasing trend for both women and men (Fig. 1). On average, the number of domestic violence calls reporting violence against men before the start of the strict lockdown increased by 69.6 calls per month (95% CI: 55.1 to 84.2) and domestic violence calls reporting violence against women increased by 224.4 calls per month (95% CI: 170.5 to 278.4) (Table 2).

**Table 2:** Summary of the model parameters and estimates.

Measure of interest	Overall		Children		Adults		Older adults	
	Parameter	95% confidence interval	Parameter	95% confidence interval	Parameter	95% confidence interval	Parameter	95% confidence interval
Pre-intervention trend for control	69.6	55.1 to 84.2	43.9	35.1 to 52.8	18.5	13.8 to 23.2	5.3	3.9 to 6.8
Pre-intervention trend for treatment	224.4	170.5 to 278.4	59.8	50.0 to 69.5	142.7	101.5 to 183.8	14.0	10.9 to 17.1
First-intervention trend for control	714.2	520.1 to 908.3	208.5	71.9 to 345.1	409.1	365.4 to 452.8	80.4	66.4 to 94.4
First-intervention trend for treatment	3053.4	2483.9 to 3622.9	328.6	170.5 to 486.7	2396.4	1992.9 to 2799.9	234.9	210.0 to 259.8
Second-intervention trend for control	-30.5	-88.2 to 27.3	0.9	-20.9 to 22.7	-24.9	-52.4 to 2.7	-6.2	-14.3 to 2.0
Second-intervention trend for treatment	-238.37	-465.2 to -11.6	-20.6	-53.7 to 12.6	-195.6	-365.7 to -25.5	-20.4	-39.4 to -1.4
Difference pre-intervention versus first-intervention: control	644.6	449.3 to 839.9	164.6	27.6 to 301.5	390.6	346.1 to 435.1	75.1	61.0 to 89.2
Difference pre-intervention versus first-intervention: treatment	2829.0	2251.7 to 3406.3	268.8	108.8 to 428.8	2253.7	1847.6 to 2659.9	220.9	195.9 to 245.9
Difference first versus second-intervention: control	-744.7	-931.4 to -557.9	-207.6	-339.5 to -75.8	-434.0	-481.6 to -386.3	-86.6	-101.3 to -71.8
Difference first versus second-intervention: treatment	-1489.3	-1862.8 to -1115.9	-415.3	-678.9 to -151.6	-867.9	-963.3 to -772.6	-173.1	-202.6 to -143.7

**Notes.** The pre-intervention trend for control refer to the $\beta_{1}$ parameter in Equation (1). The pre-intervention trend for treatment was calculated as $\beta_{5}+\beta_{1}$. First -intervention trend for control was measured as $\beta_{1}+\beta_{3}$. First-intervention trend for treatment was estimated as $\beta_{1}+\beta_{3}+\beta_{5}+\beta_{7}$. Second-intervention trend for control was estimated as $\beta_{1}+\beta_{3}+\beta_{9}$. Second-intervention trend for treatment was calculated as $\beta_{1}+\beta_{3}+\beta_{5}+\beta_{7}+\beta_{9}+\beta_{11}$. Difference pre-intervention versus first intervention for the control group refers to $\beta_{3}$ in Equation (1). Difference pre-intervention versus first intervention for the treatment group was measured as $\beta_{3}+\beta_{7}$. Difference first versus second intervention for the control group refers to $\beta_{9}$ in Equation (1). Difference first versus second intervention for the treatment group was calculated as $\beta_{9}+\beta_{11}$.

Immediately following the introduction of the strict lockdown, the number of domestic violence calls increased dramatically, especially for violence against women (Fig. 1). On average, immediately after the introduction of the lockdown, the number of domestic violence calls reporting violence against women was 3053.4 per month (95% CI: 2483.9 to 3622.9) (Table 2). Compared to the previous pre-intervention trend, an additional 2829 domestic violence calls per month were reported (95% CI: 2251.7 to 3406.3). On the other hand, immediately after the introduction of the lockdown, the number of domestic calls reporting violence against men was -744.7 per month (95% CI: -931.4 to -557.9). Compared to the previous pre-intervention trend, an increase of 644.6 monthly domestic violence calls per month was reported (95% CI: 449.3 to 839.9) (Table 2).

However, the increasing trend shown during the strict lockdown for women and men switched to a decreasing trend when the strict lockdown was relaxed (Fig. 1), albeit with levels hat were not as low as those reported in the pre-intervention period (that means previous to the COVID-19 pandemic). On average, during the second intervention period the number of domestic calls reporting violence against women reduced by -238.4 per month (95% CI: -465.2 to -11.6). For violence against men, during the second intervention period the number of domestic violence calls reduced, on average, to -30.5 calls per month (95% CI: -88.2 to 27.3), although this reduction was not statistically significant (Table 2).

**Fig. 2:**
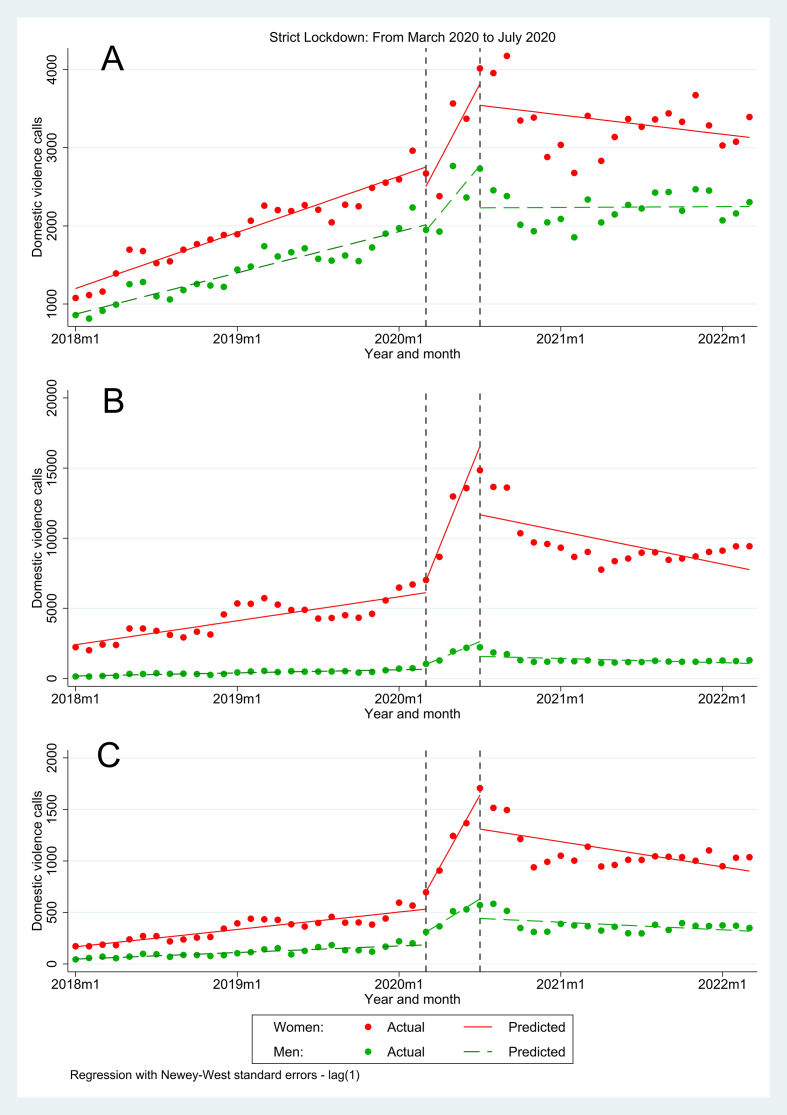
Interrupted Time Series: Domestic violence calls from January 2018 to March 2022. A. Survivors from 0 to 17 years old; B. Survivors from 18 to 59 years old. C. Survivors 60 or more years old.

When we analyzed subgroups of individuals, we found similar results and patterns as those reported in the overall population (see Panel A, Panel B and Panel C of Fig. 2). A summary of the trends during the pre-intervention period, after the intervention period and after the second intervention period is shown in Table 2.

## 4 Discussion

### 4.1 Main findings

Our results confirm that women had a greater increase in the reporting of domestic violence through calls to Línea 100 immediately after the initiation of strict lockdown and after lockdown compared to their male counterparts. This same pattern was observed in the reporting of domestic violence among children, adults, and older adults. The slope of the number of domestic violence calls after lockdown has not returned to its pre-pandemic values, especially in domestic violence against women.

### 4.2 Comparison with previous studies

The increase in the reporting of domestic violence after the onset of confinement and after confinement was found to be greater among females compared to males in all age groups. This result is similar to that reported in a SR that evaluated the effect of COVID-19 confinement on the reporting of domestic violence incidents. This SR included 18 studies conducted in the United States (n = 12), Mexico (n = 1), Italy (n = 1), Sweden (n = 1), Australia (n = 1), Argentina (n = 1) and India (n = 1) and evaluated domestic violence incidents weeks or months after the start of confinement. Through a meta-analysis, they reported a 7.86% increase in the average number of domestic violence reports after lockdown compared to the pre-pandemic period (Piquero et al., 2021). Another SR that included 32 studies conducted in various regions of the world (Europe, Asia, Africa and America), found an increase in the reporting of domestic violence incidents in all the individual studies included after lockdown compared to the pre-pandemic period, which is similar to our findings (Kourti et al., 2023). However, the authors of this SR noted that the reporting of domestic violence in children decreased during the same period (Kourti et al., 2023). This finding is similar to that reported in a study conducted in Zimbabwe (which was not included in this SR), in which the authors noted a decrease in telephone reporting of child abuse and maltreatment during the pandemic period, which is dissimilar to our finding (Cerna-Turoff et al., 2022). Although telephone reporting of domestic violence may have decreased in children in this study, the authors mentioned that this decrease would not explain a real decrease in the prevalence of child abuse (Cerna-Turoff et al., 2022). Thus, our findings could be attributed to various stressor events such as school closings, parental job loss, and a lack of national action, which could be related to an increase in child violence, as described in previous studies (Irwin et al., 2022; Lawson et al., 2020).

On the other hand, although both SRs showed an increasing pattern in the reporting of domestic violence, these results only provide a picture of the general population, without considering the sex of the survivor (Kourti et al., 2023; Piquero et al., 2021). In this sense, our finding of increased reporting of violence in women could be attributed to a decrease in the action of support services because of the lockdown instituted by governments, as previously reported (Evans et al., 2020). In addition, the Office for National Statistics in England and Wales showed a 6% increase in domestic violence crimes during the pandemic, with the majority of survivors being female (Stripe, 2020). These figures expose a social and health problem that has been considered a shadow pandemic by the United Nations (UN) (United Nations Women, 2021), in which women have experienced increased acts of violence, including fatal consequences because of the stay-at-home order. This social problem has led governmental and non-governmental institutions such as the UN along with local governments, justice departments, police and health departments in countries to formulate strategies to create a safe environment and prevent women from suffering higher levels of intimate partner violence (United Nations Women, 2021).

In Peru, a study that used data from telephone calls to Línea 100 found a 48% increase in the incidence rate of calls about domestic violence against women between April and July 2020 compared with the same months in 2019 (Agüero, 2021). However, these results were considered only four months after strict lockdown was instituted, which would not allow determining whether these figures underwent an increase or decrease in subsequent months. In this sense, our study indicates that after strict lockdown, the number of telephone reports of domestic violence has not returned to pre-pandemic values, prompting governmental institutions to take immediate action on this difficult-to-manage social problem. Specifically, the MIMP implemented the “National Strategy for the Implementation of the Specialized National System of Justice for the Protection and Punishment of Violence Against Women and Family Group Members 2021–2026”, whose objective is to establish mechanisms, measures and policies to prevent, attend and protect survivors of violence, as well as to provide punitive measures to aggressors to guarantee a life free of violence (Ministerio de la Mujer y Poblaciones Vulnerable, 2021b).

### 4.3 Implications for public health and society

Although telephone reports of violence against Peruvian children increased during the beginning of the COVID-19 pandemic (Hernández et al., 2022), it has been reported that the seven INSPIRE strategies that seek to eradicate child violence in all its forms have been partially implemented, including the acronyms N and I that refer to Norms and Values, and Economic Strengthening and Income, respectively, have not been implemented in Peru (World Health Organization, 2020), thereby limiting the protection of children through national action plans and financial support to mothers. Thus, the government must ensure compliance with the seven strategies in order to reduce the short- and long-term consequences for individuals and society. Likewise, the Peruvian government has recently established comprehensive interventions to prevent violence against women in the community, education, labor and unions, legal and media environments through the strategy " Mujeres libres de violencia” (“Women free of violence” in English) because more than half of Peruvian women experienced violence during the pandemic (Ministerio de la Mujer y Poblaciones Vulnerable, 2021c). In this sense, this strategy should not only strengthen economic, educational and sexual spheres of women, but also their mental and emotional health, which have been factors that have increased their susceptibility to domestic violence (United Nations Women, 2021). Finally, there are several strategies that prevent violence against older adults. However, this population group has been little mentioned in strategies against violence, and therefore, government institutions should redouble their efforts to strengthen and establish comprehensive strategies that have been included as part of a proposed national agenda for the care and protection of older adults (Mesa de Concertación para la Lucha contra la Pobreza, 2023).

### 4.4 Strengths and limitations

One of the strengths of our study is the inclusion of the period after strict lockdown, which provides a current picture of pandemic telephone reporting and indirectly of domestic violence cases suffered by Peruvian household members. In addition, we use nationwide data that have been collected by the only Peruvian institution (MIMP) that seeks to prevent domestic violence in all its forms.

Nevertheless, the study should be considered with some limitations. First, the telephone reports on domestic violence may have presented recording errors on the part of the operator receiving the call. Second, these reports may not include rural communities that do not have access to adequate telephone signal within their homes. Third, the telephone calls were made in Spanish, Quechua and Aymara; however, there are rural communities in the highlands and jungle that communicate in other dialects such as Ashaninka, Awajún/Aguaruna, Matsiguenka/Machiguenga, Shipibo - Konibo, or Shawi/Chayahuita (Instituto Nacional de Estadística e Informática, 2018). However, this type of native languages is observed in a low number of participants due to the fact that these people have little access to basic services and telephony, as well as the possible lack of knowledge of services provided by the state such as Línea 100. Fourth, there is a possibility that people may make false calls, or that the information provided is not true; however, Línea 100 has established procedures not to register this type of call, and the telephone operators try to ensure that the data collected are consistent as much as possible (Ministerio de la Mujer y Poblaciones Vulnerable, 2021a). Fifth, these data may have been underreported because confinement may not provide an adequate or safe space or setting to report violent acts or, in the case of the report of violence by men, there are some studies where they mention that they seek help from a variety of resources, more typically from informal resources, such as family, friends and the Internet, and there are also factors to seek help related to the social stigma and negative emotional impact (Douglas & Hines, 2011; Machado et al., 2017; Tsui et al., 2010). In addition, age and individual characteristics could be other predisposing factors for the underreporting of domestic violence. In fact, children, older adults and people with disabilities would have greater difficulty in reporting these facts due to their functional or economic dependence on the perpetrator. Sixth, it is necessary to mention that the Línea 100 data may not capture the totality of domestic violence incidents, as it is possible that some cases are not reported, and that people seek help from alternative sources such as going directly to the police station at report the case. Seventh, our study is a repeated cross-sectional analysis that only allows us to identify trends in domestic violence calls before, during and after the lockdown, but does not provide a causal explanation. Finally, the research was limited to a single helpline in one country, and considering the topic addressed, it may limit the direct applicability of the findings to other regions or countries with different sociocultural contexts, levels of support systems, or confinement measures. Nevertheless, our findings provide a preliminary overview of the cases of violence experienced by the Peruvian population.

## 5 Conclusions

Domestic violence against women, regardless of age, continues to be a serious social-health problem that has seen an increase in telephone reports during strict lockdown. It is troubling to note that, despite the end of confinement, the numbers have not returned to pre-pandemic levels. These data highlight the need to place special emphasis on the disproportionate impact that domestic violence has on women, requiring urgent attention and a comprehensive response. In this sense, government institutions should promote and strengthen strategies that prevent domestic violence in all age groups in order to reduce this social problem that continues to be neglected in Peru.

## Ethics approval and consent to participate

All data analyzed in the current research were obtained from publicly available datasets. Data are freely available at https://portalestadistico.aurora.gob.pe/bases-de-datos-2022/and does not contain any personally identifiable information.

## Ethical statement

Since the study involved analyzing aggregated secondary data that is publicly available (https://portalestadistico.aurora.gob.pe/bases-de-datos-2022/) and does not allow for the identification of the evaluated participants, it did not necessitate the approval of an ethics committee.

## Consent for publication

Not applicable.

## Funding

This study did not receive any specific grant from any funding agencies.

## Authors’ contributions

AHV: Conceptualization, Supervision, Methodology, Investigation, Data curation, Formal analysis, Writing- Original draft preparation, Writing- Reviewing and Editing. RVF: Investigation, Validation, Writing- Original draft preparation, Writing- Reviewing and Editing. ETL: Investigation, Writing- Reviewing and Editing. CRR: Supervision, Methodology, Investigation, Validation, Formal analysis, Writing- Original draft preparation, Writing- Reviewing and Editing. All authors read and approved the final manuscript. All authors read and approved the final manuscript.

## Declaration of competing interest

The authors have no conflicts of interest to declare.

## Data Availability

Data are available at https://portalestadistico.aurora.gob.pe/bases-de-datos-2022/.
